# Reproducibility of AI in Cephalometric Landmark Detection: A Preliminary Study

**DOI:** 10.3390/diagnostics15192521

**Published:** 2025-10-05

**Authors:** David Emilio Fracchia, Denis Bignotti, Stefano Lai, Stefano Cubeddu, Fabio Curreli, Massimiliano Lombardo, Alessio Verdecchia, Enrico Spinas

**Affiliations:** 1 Department of Surgical Sciences, Postgraduate School in Orthodontics, University of Cagliari, 09124 Cagliari, Italy; david.fracchia@gmail.com (D.E.F.); denis.bignotti3@gmail.com (D.B.); stefano87lai@gmail.com (S.L.); stefanocubeddu98@gmail.com (S.C.); f.curreli6@studenti.unica.it (F.C.); info@massimilianolombardo.com (M.L.); 2 Orthodontics Division, Instituto Asturiano de Odontologia, Universidad de Oviedo, 33006 Oviedo, Spain

**Keywords:** artificial intelligence, cephalometry, anatomic landmarks, orthodontics, corrective, image processing, computer-assisted

## Abstract

**Objectives**: This study aimed to evaluate the reproducibility of artificial intelligence (AI) in identifying cephalometric landmarks, comparing its performance with manual tracing by an experienced orthodontist. **Methods**: A high-quality lateral cephalogram of a 26-year-old female patient, meeting strict inclusion criteria, was selected. Eighteen cephalometric landmarks were identified using the WebCeph software (version 1500) in three experimental settings: AI tracing without image modification (AInocut), AI tracing with image modification (AI-cut), and manual tracing by an orthodontic expert. Each evaluator repeated the procedure 10 times on the same image. X and Y coordinates were recorded, and reproducibility was assessed using the coefficient of variation (CV) and centroid distance analysis. Statistical comparisons were performed using one-way ANOVA and Bonferroni post hoc tests, with significance set at *p* < 0.05. **Results**: AInocut achieved the highest reproducibility, showing the lowest mean CV values. Both AI methods demonstrated greater consistency than manual tracing, particularly for landmarks such as Menton (Me) and Pogonion (Pog). Gonion (Go) showed the highest variability across all groups. Significant differences were found for the Posterior Nasal Spine (PNS) point (*p* = 0.001), where AI outperformed manual tracing. Variability was generally higher along the X-axis than the Y-axis. **Conclusions**: AI demonstrated superior reproducibility in cephalometric landmark identification compared to manual tracing by an experienced operator. While certain points showed high consistency, others—particularly PNS and Go—remained challenging. These findings support AI as a reliable adjunct in digital cephalometry, although the use of a single radiograph limits generalizability. Broader, multi-image studies are needed to confirm clinical applicability.

## 1. Introduction

Artificial Intelligence (AI), a term first coined by John McCarthy at the Dartmouth Conference in 1956 [1], has undergone significant evolution in both theory and application. Over the last decade, its integration into the medical and dental domains, particularly orthodontics, has attracted growing interest. The implementation of AI-based technologies aims to enhance reproducibility, reduce human error, and improve diagnostic precision, especially in radiographic interpretation, as also highlighted in previous studies emphasizing the importance of standardized and reproducible assessment methods in dentistry [2,3,4]. Cephalometry remains a cornerstone of orthodontic diagnosis and treatment planning. Accurate identification of cephalometric landmarks is essential for assessing skeletal discrepancies, planning interventions, and evaluating treatment outcomes. However, manual tracing is inherently subject to inter- and intra-operator variability, which may affect the reliability of clinical decisions [5,6,7]. To address these challenges, AI-driven solutions have been increasingly proposed to standardize cephalometric analyses. In 2014, international AI competitions such as those promoted by the IEEE International Symposium on Biomedical Imaging (ISBI) accelerated the development of automated tools for landmark detection [8]. Among the earliest and most prominent methods, Convolutional Neural Networks (CNNs) have proven highly effective in locating cephalometric landmarks on lateral radiographs with remarkable speed and accuracy [9]. The YOLOv3 architecture has gained widespread use due to its ability to perform real-time object detection efficiently [10]. Multiple studies have validated that AI-powered algorithms can reach, and in some cases exceed, the precision achieved by experienced orthodontists [11,12]. Furthermore, when combined with human oversight, AI systems have been shown to enhance diagnostic consistency among less experienced clinicians, as illustrated by Panesar et al. [13], who reported significant improvements in accuracy when AI-assisted workflows were adopted in clinical training. Recent advances in deep learning have seen a shift from CNN-based models to Transformer-based architectures, especially Vision Transformers (ViTs). Unlike traditional approaches that rely on heatmap regression, ViTs predict landmark coordinates directly through self-attention mechanisms. Laitenberger et al. [14] reported that a ViT-based system significantly reduced the average radial error by more than 2 mm compared to prior CNN-based methods, particularly in large, weakly annotated datasets. In parallel, deep learning applications have expanded to soft tissue analysis. Han et al. [15] developed a CNN model capable of classifying lip thickness directly from cephalometric images, achieving over 90% diagnostic accuracy. These capabilities open new avenues for personalized treatment planning and the integration of morphological parameters into orthodontic decision-making. The benefits of AI are not limited to clinical diagnosis. In orthodontic education, AI-enhanced platforms have shown to accelerate learning and improve precision among novice students. Lin et al. [16] emphasized that real-time visual feedback from AI systems significantly enhances landmark tracing skills while reducing both instructional workload and inter-operator variability. Nonetheless, despite these technological advances, concerns regarding reliability and clinical applicability remain. Studies such as those by Guinot-Barona et al. [17] have identified substantial discrepancies between AI and expert tracings for certain landmarks, reaffirming the necessity for ongoing professional oversight, particularly in borderline or complex anatomical cases. Independent validations have further contributed to assessing the robustness of commercial AI solutions. WebCeph, a widely used cloud-based platform, has shown reliable performance in clinical settings, with studies like Lee et al. [18] reporting a mean error under 1.3 mm and 89% accuracy within 2 mm. However, landmarks such as PNS, Orbitale, and Articulare showed greater variability. Similarly, Kartbak et al. [19] highlighted that while AI performs well overall, differences across anatomical regions and clinical contexts reinforce the need for human oversight. Finally, studies by Gallardo-Lopez [20] underscored the inherent variability in manual landmark identification, revealing errors greater than 2 mm even among trained professionals. These findings further support the value of AI tools in reducing subjectivity and enhancing reproducibility. The primary objective of this preliminary study was to evaluate the reproducibility of cephalometric landmark localization using an artificial intelligence (AI) model and to compare its accuracy and consistency with the manual localization of the same landmarks performed by a board-certified orthodontist. This comparison was carried out under standardized diagnostic conditions, with the aim of providing an initial assessment of the potential applicability of AI-based methods in routine orthodontic diagnostic workflows.

## 2. Materials and Methods

This investigation was designed as a preliminary methodological reliability/agreement study, reported in accordance with the Guidelines for Reporting Reliability and Agreement Studies (GRRAS) [21] and the Checklist for Artificial Intelligence in Medical Imaging (CLAIM) [22]. All procedures were conducted in compliance with the ethical standards of the Declaration of Helsinki [23]. The study protocol was reviewed and approved by the Ethics Committee of the Surgical Sciences Department, University of Cagliari, Italy (PG/2021/11308). The data source consisted of the orthodontic database of the Dental Clinic, Section of Orthodontics, University of Cagliari. After establishing the inclusion criteria, a single lateral cephalometric radiograph was randomly selected. The inclusion criteria were: absence of cranio-dento-maxillofacial syndromes or conditions that could significantly affect the anatomical structures; absence of metallic artifacts; correct patient positioning in the cephalostat; and adequate radiographic resolution. On this basis, one high-quality lateral cephalogram was selected from a 26-year-old female patient scheduled for orthodontic treatment at the University of Cagliari’s Dental Clinic. Written informed consent was obtained from the patient prior to inclusion. The radiograph was acquired using a MyRay Hyperion X5 3D PAN-CEPH X-ray unit, exported in .jpg format, and analyzed with WebCeph software (version 1500), which enables both artificial intelligence-based and manual cephalometric tracing. For the manual tracings, a board-certified orthodontist with 25 years of clinical experience performed all landmark identifications to ensure consistency and reliability. Cephalometric landmarks were identified on the same image using both the AI-assisted method and the manual method.

### 2.1. Cephalometric Landmarks

A total of 18 cephalometric landmarks were selected for analysis, based on their clinical relevance in orthodontics:

- Point (A);
- Anterior Nasal Spine (ANS);
- Articulare (Ar);
- Point (B);
- Gnathion (Gn);
- Gonion (Go);
- Lower Incisal Tip (L1);
- Lower Lip (LL);
- Menton (Me);
- Nasion (NA);
- Soft Tissue Pogonion (ST-Pog);
- Orbitale (Or);
- Posterior Nasal Spine (PNS);
- Porion (Po);
- Pogonion (Pog);
- Subnasale (SN);
- Upper Incisal Tip (U1);
- Upper Lip (UL).

**Figure 1:**
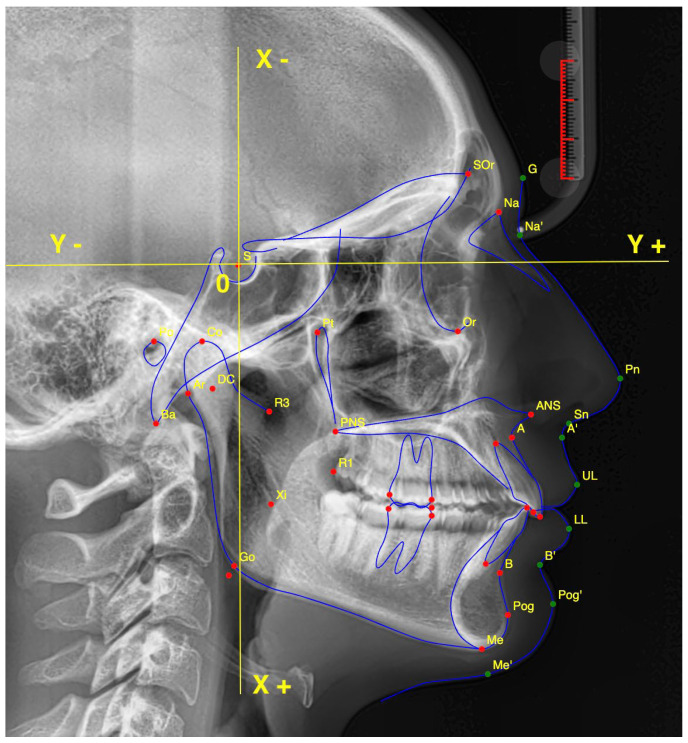
Localization of 18 cephalometric landmarks with inclusion of the S point as the central reference.

Additionally, the S-Point (S)—representing the center of the sella turcica—was not included among the 18 analyzed points but was used exclusively as the origin of the Cartesian coordinate system (X and Y axes) for positional analysis (Figure 1). This choice was dictated by a technical feature intrinsic to the cephalometric analysis software employed in this study (WebCeph, Seongnam, Republic of Korea), which automatically registers the coordinates of each cephalometric landmark using the S-Point as the (0,0) reference. From this origin, the coordinates of all other cephalometric landmarks can subsequently be determined.

### 2.2. Study Design and Experimental Groups

**Figure 2:**
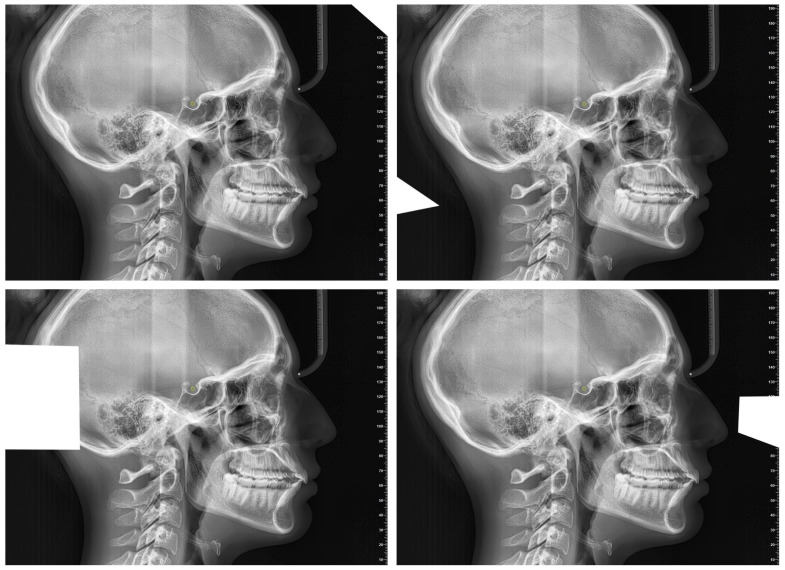
Examples of Cropped and Resized Images.

Each evaluator repeated the landmark identification procedure 10 times on the same image. The three experimental groups were:

- Artificial Intelligence Cephalometric Tracing—No Image Modification (AInocut):
  AI-assisted identification of the 18 landmarks was performed on the original, unmodified image. The procedure was repeated 10 times, modifying only the patient identification data between trials to avoid potential duplication or memory effects by the software.

- Artificial Intelligence Cephalometric Tracing—With Image Modification (AIcut):
  The same AI model was used to detect landmarks, but the image was intentionally resized or cropped in each of the 10 repetitions. This aimed to test the AI’s robustness and precision under altered input conditions (Figure 2). The image cropping was intended to alter the grayscale values at the image margins, which represent one of the parameters used by the AI to orient itself within the radiograph. Importantly, the introduction of these crops did not in any way affect the anatomical structures relevant for landmark localization.

- Experienced Operator Manual Cephalometric Tracing (Oper):
  The same 18 landmarks were manually traced by the expert operator 10 times, with 15-day intervals between repetitions to minimize memory bias and assess intra-operator reproducibility.

### 2.3. Data Processing

From each tracing, the X and Y coordinates of the 18 landmarks were extracted, using S-Point (S) as the Cartesian origin. This yielded 36 coordinate values per tracing (18 points × 2 dimensions), and a total of 1080 coordinate values across all three groups (3 groups × 10 repetitions × 18 points × 2). For each landmark, the mean predicted position (centroid) was calculated across the 10 repetitions, and the Euclidean distance of each individual point from this mean was computed. These distances were used as reproducibility indicators for statistical evaluation.

### 2.4. Statistical Analysis

To evaluate the reproducibility of cephalometric landmark identification across the different evaluators and experimental conditions, a structured statistical analysis was carried out. Initially, descriptive statistics were used to summarize the data, providing key indicators such as mean, standard deviation, minimum, maximum, and median for each continuous variable. For categorical variables, absolute and relative frequencies were also calculated. These descriptive results offered a comprehensive overview of the variability associated with each method. To further assess the consistency of landmark identification, the coefficient of variation (CV) was calculated for each landmark and evaluator. This allowed for the quantification of relative variability in point placement, serving as a key metric in evaluating the internal consistency of each method. In order to determine whether there were statistically significant differences in reproducibility between the three groups (AI with unmodified images, AI with modified images, and manual tracing by an expert), a one-way analysis of variance (ANOVA) was performed. This test compared the average distances of each point from its respective mean (centroid) across the 10 repetitions. When ANOVA indicated significant differences, post hoc pairwise comparisons were conducted using the Bonferroni correction, which adjusts the significance threshold to account for the increased risk of false positives due to multiple testing. Throughout the analysis, a significance level of *p* < 0.05 was adopted. Differences were considered statistically significant only when the *p*-value was less than 0.05, ensuring a 95% confidence level in the results.

## 3. Results

### 3.1. Analysis of Coordinate Variability

#### 3.1.1. Coefficient of Variation (CV) per Single Point

**Figure 3:**
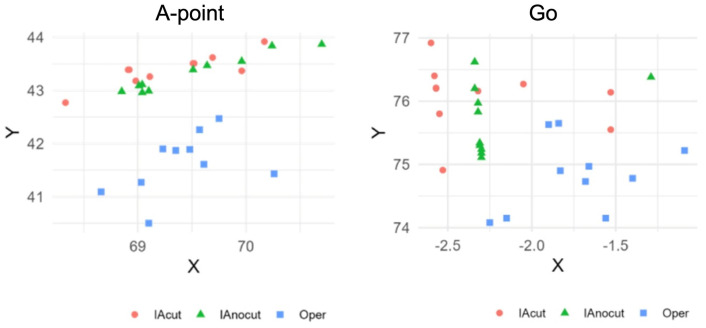
Scatter plots showing representative examples of point distribution on the Cartesian plane: Point A and Go.

The CV was used to quantify the relative variability of the X and Y coordinates. Lower CV values indicate higher precision and, consequently, better reproducibility. CV values were calculated for each parameter and for each Cartesian axis (X, Y), as well as the arithmetic mean of the two. In the scatter plots, the distribution of the predicted points across the Cartesian plane by the different evaluators for each parameter is shown (Figure 3). The AI models (both IAcut and IAnocut) generally exhibited lower CV values compared to the human operator, indicating greater consistency in the predictions.

#### 3.1.2. Overall Mean Coefficient of Variation by Evaluation Method

Table 1 describes the reproducibility of measurements by evaluators for each parameter. The unmodified AI model (IAnocut) showed the highest overall reproducibility, as indicated by the lowest overall mean CV. Variability along the X-axis was consistently higher than along the Y-axis, suggesting greater difficulty in predicting the horizontal component.

#### 3.1.3. Parameters with Highest and Lowest Variability

In Table 2, the mean coefficient of variation (CV) for each parameter is presented, calculated as the average across the different evaluators. Reproducibility was very high for landmarks such as Me and Pog, while it was significantly lower for point Go, likely due to imaging artifacts or anatomical ambiguity.

### 3.2. Analysis of Distances to the Centroid

This analysis aims to quantify the overall geometric consistency of the predictions: smaller distances indicate greater internal coherence of the measurement system.

#### Analysis of Variance

**Figure 4:**
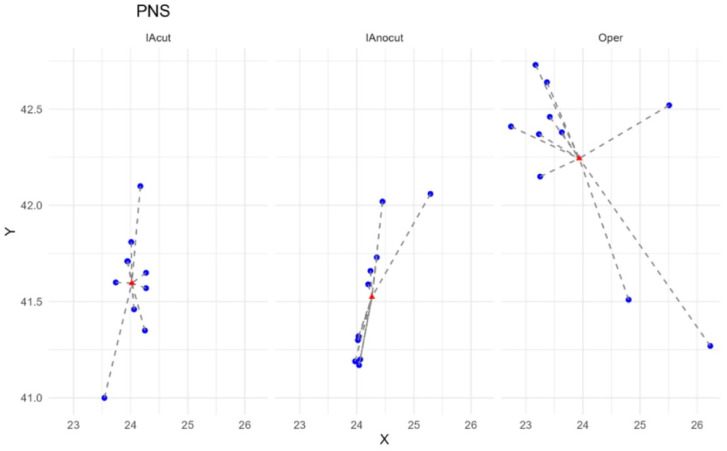
Distance to the centroid of the predicted PNS point for each evaluator.

Out of the 18 cephalometric points analyzed, only the PNS point showed statistically significant differences between methods (*p* = 0.001). For the remaining 17 points, differences in distances to the centroid were not statistically significant (*p* > 0.05), neither in the ANOVA nor in the Bonferroni post hoc tests. In Figure 4, the variability observed in the localization of the PNS point across the different evaluation methods can be appreciated. The differences between the manual method and both AI approaches were statistically significant (IAcut vs. Operator: *p* = 0.002; IAnocut vs. Operator: *p* = 0.007), indicating that the manual method exhibits significantly greater variability for the PNS point, thus confirming its lower reproducibility. In all other cases, the differences in variability between measurement methods did not reach statistical significance. However, the manual method tended to produce greater distances in some instances. For certain landmarks, such as Na and LowerLip, the differences came close to significance (e.g., Na: *p* = 0.099), suggesting a possible trend toward lower reproducibility.

## 4. Discussion

Cephalometric analysis remains a fundamental diagnostic tool in orthodontics and maxillofacial surgery. With the increasing adoption of digital technologies, AI-powered platforms such as WebCeph (Seongnam, Republic of Korea) have emerged to support automated landmark identification on lateral cephalograms. Although these tools promise to enhance clinical efficiency, questions persist regarding their accuracy and reproducibility when compared to conventional manual methods. The analysis of the results from our study clearly highlights the superior reproducibility and spatial consistency of cephalometric landmark identification when performed by artificial intelligence, compared to manual tracing by a human operator. In particular, the coefficient of variation (CV) used as a measure of relative variability consistently yielded lower values across both of our deep neural network models, IAcut and IAnocut, than for the manual method. Notably, IAnocut the model applied without predefined image cropping exhibited the lowest overall mean CV, suggesting that artificial intelligence can maintain stable predictions even when provided with less standardized radiographic inputs. Manual landmark identification is known to exhibit high intra- and inter-operator variability, with errors potentially exceeding 2 mm for more complex points such as Gonion and Orbitale [20]. This underscores the usefulness of AI in reducing subjectivity, ensuring greater diagnostic consistency regardless of the operator’s level of experience. In this study, the same radiograph was used consistently across all evaluations to minimize variability due to anatomical, demographic, or pathological factors such as age, sex, type of malocclusion, or craniofacial anomalies which are known to influence AI performance [12,24,25]. This methodological choice allowed the isolation of the evaluation method (manual vs. automatic) without interference from inter-subject heterogeneity. A gold standard reference was also included to distinguish between AI-specific errors and those resulting from anatomical ambiguity or intrinsic limitations of automated detection. The IAcut approach [15] involved providing images that were cropped differently at each prediction instance, intentionally introducing variability to stress-test the model under non-standard conditions. Consistently with previous findings, our results indicate that image preprocessing steps such as cropping or resizing can negatively affect the spatial accuracy of AI landmark detection. The superior stability of the IAnocut model suggests that deep neural networks trained on more variable or less constrained inputs may exhibit greater robustness when applied to non-standardized clinical images. This setup aimed to simulate the operational variability encountered in clinical practice, as well as to assess the algorithm’s sensitivity to changes in visual context and image scale factors known to affect landmark detection performance [26]. In line with recent studies, such as that by Laitenberger et al. [14], the robustness of AI models has been attributed not only to the architecture employed but also to the prediction strategy adopted. Models based on direct coordinate prediction have proven to be more stable than heatmap-based methods, particularly when dealing with non-standardized input conditions. If the AI was trained on standardized images with well-defined borders, altering the visible region could compromise localization accuracy, especially for landmarks that are highly context-dependent (e.g., Go or Na), as opposed to more isolated points like Me or Pog. Previous studies have shown that image pre-processing steps like resizing can reduce the validity of AI predictions [27]. Despite this artificial complexity, the AI models outperformed the human operator. This finding aligns with previous work by Hwang et al. (2020), who reported that artificial intelligence could surpass the accuracy of cephalometric landmark placement by clinical experts [28]. These results are consistent with those of Lin et al. [16], who observed that the use of AI systems and digital software streamlines the cephalometric diagnostic process and can significantly reduce technical variability among operators. However, reliance on automated systems requires adequate critical training to avoid passive tool usage and to ensure the preservation of essential clinical competencies. Notably, variability along the X-axis was generally greater than along the Y-axis, consistent with the challenges of horizontal landmark localization, which may stem from anatomical superimpositions, image artifacts, or spatial resolution constraints [29]. Results also revealed significant heterogeneity in reproducibility among different landmarks. Me and Pog showed minimal variability, while Go demonstrated pronounced instability, with CV values exceeding 9. Interestingly, although the centroid-based geometric analysis revealed PNS as the most statistically significant point showing greater coherence with AI methods compared to manual tracing the coefficient of variation analysis identified PNS as the landmark with the highest average variability across evaluators. This apparent contradiction highlights an important nuance: while PNS predictions may tend to cluster more consistently around a centroid in AI-based methods, the inter-method variability remains considerable, likely reflecting intrinsic anatomical ambiguity or radiographic limitations in defining this structure. The systematic review by Gallardo-Lopez [20] also identified PNS as one of the most challenging landmarks in terms of repeatability, regardless of the AI model used, suggesting the need for specific diagnostic protocols for landmarks with high uncertainty. These findings underscore the complexity of accurately and reliably identifying certain landmarks, even with advanced algorithms. These findings are consistent with the existing literature reporting that AI can achieve comparable or even superior performance to human experts [28,29]. Hendrickx et al. [30] noted that most 2D cephalometry studies using AI achieved over 80% accuracy within a 2 mm threshold, generally considered clinically acceptable [31]. Our model demonstrates performance comparable to that reported by Han et al. [15], who achieved a mean MRE of 0.97 mm and an SDR of 95.4% within 2 mm using a CNN-based architecture applied to over 1000 cephalograms. However, their approach also included the classification of lip thickness, an interesting extension aimed at integrating automated landmarking with clinical decision-making processes. Nevertheless, it is essential to recognize that reproducibility does not guarantee accuracy: a model may be highly consistent yet systematically misplace landmarks if trained on non-representative data. Moreover, AI performance may decline under specific clinical conditions. For instance, Tanikawa et al. [25] reduced landmarking accuracy in patients with cleft palate, suggesting that algorithmic generalizability [15] remains a critical issue. As previously reported in a recent meta-analysis AI accuracy may drop to as low as 65% in patients with craniofacial dysmorphisms or in radiographs containing orthodontic appliances. These findings underscore the necessity for external validation protocols tailored to complex clinical scenarios, where AI models are more likely to encounter visual ambiguities and non-representative anatomical features. Thus, validating AI outputs on independent datasets remains critical [27], especially given the potential for overfitting when evaluation is conducted on data similar to the training set [26]. Despite the promising performance of platforms such as WebCeph (Seongnam, Republic of Korea), a level of clinical subjectivity in analysis and treatment planning remains irreplaceable. Some authors suggest that incorporating minor manual corrections to AI-generated predictions may further enhance final outcomes [28], pointing toward a hybrid model that combines automation with expert oversight. This “hybrid” strategy is currently being proposed as a best practice in real-world clinical settings, where the synergy between AI and human intervention allows for maximized efficiency without compromising precision [20]. Overall, the findings of this study support the use of artificial intelligence as a reliable auxiliary tool in digital cephalometry. However, it is essential to continue testing AI models under variable and realistic conditions, ensuring that algorithmic performance remains robust and clinically valid across different contexts while always maintaining expert control over diagnostic and therapeutic decisions [32,33]. This study has several limitations that warrant consideration. First, the repeated analysis of the same radiograph may have introduced a source of bias, as both the AI system and the human operator could become conditioned by prior landmark placements across multiple iterations. This effect may have led to an artificial reduction in observed variability, potentially masking true differences in reproducibility. A more robust assessment would benefit from the use of multiple, independent radiographs to better account for both intra- and inter-image variability.

Second, the cephalometric software may apply internal preprocessing procedures—such as automatic resizing, normalization, or contrast enhancement—that are not visible to the end user but may influence the precision of landmark localization and compromise the standardization of input conditions. These technical aspects, along with the study design, should be carefully considered when interpreting the results and planning future validation efforts.

Although the data reveal promising trends, the current findings are based on a limited sample and a single-image design. To enhance the reliability and generalizability of AI-based landmark identification, future studies should include larger and more diverse datasets encompassing a wider range of anatomical and imaging conditions. Incorporating multi-image and multi-operator protocols would allow for a more accurate assessment of reproducibility and reduce potential biases linked to individual operators or specific radiographs. Moreover, including patients with varied demographic characteristics—such as age, skeletal patterns, and ethnic backgrounds—would further strengthen the clinical relevance and applicability of AI tools in cephalometric analysis.

## 5. Conclusions

This preliminary study assessed the reproducibility of AI-based cephalometric landmark identification by comparing two automated methods—one using standardized images and the other incorporating image modifications—with manual tracing by an experienced operator. The AI models, particularly when no image alterations were present, demonstrated lower variability and greater consistency in landmark localization compared to the manual method. While certain landmarks, such as Me and Pog, exhibited high reproducibility, others—most notably PNS—showed considerable variability, underscoring both anatomical complexity and methodological limitations. Although the results are promising, they are based on a single-image design and a limited dataset. Therefore, while AI systems may serve as a potentially valuable adjunct in enhancing consistency in cephalometric analysis, this role should be considered preliminary. Further studies involving larger, more diverse datasets and varied clinical scenarios are essential to validate and generalize these findings for routine orthodontic practice.

## Figures and Tables

**Table 1:** Reproducibility of measurements by evaluator per parameter: mean coefficient of variation per evaluator and confidence intervals (CI).

	IAcut X	IAcut Y	IAcut (Mean)	IAnocut X	IAnocut Y	IAnocut (Mean)	Operator X	Operator Y	Operator (Mean)
**PARAMETERS** **(Mean)**	1.90	1.12	1.51	1.81	0.83	1.32	2.15	1.11	1.63
**95%CI**	(0–4.01)	(0.75–1.49)	(0.46–2.56)	(0.20–3.42)	(0.76–0.92)	(0.52–2.12)	(0–4.39)	(0.89–1.33)	(0.53–2.73)

**Table 2:** Reproducibility of measurements by evaluators per parameter: mean coefficient of variation by parameter.

	Evaluator (Mean)
**A-point**	0.87
**ANS**	0.81
**AR**	1.51
**B-point**	0.84
**Gn**	0.86
**Go**	9.25
**L1Incisal**	0.76
**LowerLip**	0.92
**Me**	0.75
**Na**	1.56
**SoftTissue**	0.77
**Or**	1.40
**UpperLip**	0.90
**U1Incisal**	0.78
**Subnasale**	0.87
**Pog**	0.75
**Po**	1.49
**PNS**	1.69

## Data Availability

Data are unavailable due to the privacy of the patients.

## References

[1] Obermeyer Z., Emanuel E.J. (2016). Predicting the Future—Big Data, Machine Learning, and Clinical Medicine. N. Engl. J. Med..

[2] Schwendicke F., Samek W., Krois J. (2020). Artificial Intelligence in Dentistry: Chances and Challenges. J. Dent. Res..

[3] Kothari S., Gionfrida L., Bharath A.A., Abraham S. (2019). Artificial Intelligence (AI) and rheumatology: A potential partnership. Rheumatology.

[4] Rawson T.M., Ahmad R., Toumazou C., Georgiou P., Holmes A.H. (2019). Artificial intelligence can improve decision-making in infection management. Nat. Hum. Behav..

[5] Baumrind S., Miller D.M. (1980). Computer-aided head film analysis: The University of California San Francisco method. Am. J. Orthod..

[6] Forsyth D.B., Shaw W.C., Richmond S., Roberts C.T. (1996). Digital imaging of cephalometric radiographs, Part 2: Image quality. Angle Orthod..

[7] Angelakopoulos N., De Luca S., Oliveira-Santos I., Ribeiro I.L.A., Bianchi I., Balla S.B., Kis H.C., Jiménez L.G., Zolotenkova G., Yusof M.Y.P. (2023). Third molar maturity index (I3M) assessment according to different geographical zones: A large multi-ethnic study sample. Int. J. Legal Med..

[8] Wang C.W., Huang C.T., Hsieh M.C., Li C.H., Chang S.W., Li W.C., Vandaele R., Marée R., Jodogne S., Geurts P. (2015). Evaluation and Comparison of Anatomical Landmark Detection Methods for Cephalometric X-Ray Images: A Grand Challenge. IEEE Trans. Med. Imaging.

[9] Ren R., Luo H., Su C., Yao Y., Liao W. (2021). Machine learning in dental, oral and craniofacial imaging: A review of recent progress. PeerJ.

[10] Park J.H., Hwang H.W., Moon J.H., Yu Y., Kim H., Her S.B., Srinivasan G., Aljanabi M.N.A., Donatelli R.E., Lee S.J. (2019). Automated identification of cephalometric landmarks: Part 1-Comparisons between the latest deep-learning methods YOLOV3 and SSD. Angle Orthod..

[11] Liu J.-K., Chen Y.-T., Cheng K.-S. (2000). Accuracy of computerized automatic identification of cephalometric landmarks. Am. J. Orthod. Dentofac. Orthop..

[12] Kim H., Shim E., Park J., Kim Y.J., Lee U., Kim Y. (2020). Web-based fully automated cephalometric analysis by deep learning. Comput. Methods Programs Biomed..

[13] Panesar S., Zhao A., Hollensbe E., Wong A., Bhamidipalli S.S., Eckert G., Dutra V., Turkkahraman H. (2023). Precision and Accuracy Assessment of Cephalometric Analyses Performed by Deep Learning Artificial Intelligence with and without Human Augmentation. Appl. Sci..

[14] Laitenberger F., Scheuer H.T., Scheuer H.A., Lilienthal E., You S., Friedrich R.E. (2025). Cephalometric landmark detection using vision transformers with direct coordinate prediction. J. Craniomaxillofac. Surg..

[15] Han M., Huo Z., Ren J., Zhu H., Li H., Li J., Mei L. (2025). Automated Landmark Detection and Lip Thickness Classification Using a Convolutional Neural Network in Lateral Cephalometric Radiographs. Diagnostics.

[16] Lin J., Liao Z., Dai J., Wang M., Yu R., Yang H., Liu C. (2025). Digital and artificial intelligence-assisted cephalometric training effectively enhanced students’ landmarking accuracy in preclinical orthodontic education. BMC Oral Health.

[17] Guinot-Barona C., Alonso Pérez-Barquero J., Galán López L., Barmak A.B., Att W., Kois J.C., Revilla-León M. (2024). Cephalometric analysis performance discrepancy between orthodontists and an artificial intelligence model using lateral cephalometric radiographs. J. Esthet. Restor. Dent..

[18] Lee H.T., Chiu P.Y., Yen C.W., Chou S.T., Tseng Y.C. (2024). Application of artificial intelligence in lateral cephalometric analysis. J. Dent. Sci..

[19] Kartbak S.B.A., Özel M.B., Kocakaya D.N.C., Çakmak M., Sinanoğlu E.A. (2025). Classification of Intraoral Photographs with Deep Learning Algorithms Trained According to Cephalometric Measurements. Diagnostics.

[20] Gallardo-Lopez E.A., Moreira L., Cruz M.H., Meneses N., Schumiski S., Salgado D., Crosato E.M., Costa C. (2025). Cephalometric Tracing: Comparing Artificial Intelligence and Augmented Intelligence on Online Platforms. Dentomaxillofac. Radiol..

[21] Kottner J., Gajewski B.J., Streiner D.L. (2011). Guidelines for Reporting Reliability and Agreement Studies (GRRAS). Int. J. Nurs. Stud..

[22] Tejani A.S., Klontzas M.E., Gatti A.A., Mongan J.T., Moy L., Park S.H., Kahn C.E., CLAIM 2024 Update Panel (2024). Checklist for Artificial Intelligence in Medical Imaging (CLAIM): 2024 Update. Radiol. Artif. Intell..

[23] Goodyear M.D., Krleza-Jeric K., Lemmens T. (2007). The Declaration of Helsinki. BMJ.

[24] Darkwah W.K., Kadri A., Adormaa B.B., Aidoo G. (2018). Cephalometric study of the relationship between facial morphology and ethnicity: Review article. Transl. Res. Anat..

[25] Tanikawa C., Lee C., Lim J., Oka A., Yamashiro T. (2021). Clinical applicability of automated cephalometric landmark identification: Part I-Patient-related identification errors. Orthod. Craniofac. Res..

[26] Raghu M., Poole B., Kleinberg J., Ganguli S., Sohl-Dickstein J. (2017). On the expressive power of deep neural networks. Proc. Mach. Learn. Res..

[27] Eche T., Schwartz L.H., Mokrane F.Z., Dercle L. (2021). Toward generalizability in the deployment of artificial intelligence in radiology: Role of computation stress testing to overcome underspecification. Radiol. Artif. Intell..

[28] Hwang H.W., Park J.H., Moon J.H., Yu Y., Kim H., Her S.B., Srinivasan G., Aljanabi M.N.A., Donatelli R.E., Lee S.J. (2020). Automated identification of cephalometric landmarks: Part 2-Might it be better than human?. Angle Orthod..

[29] Kim J., Kim I., Kim Y.J., Kim M., Cho J.H., Hong M., Kang K.H., Lim S.H., Kim S.J., Kim Y.H. (2021). Accuracy of automated identification of lateral cephalometric landmarks using cascade convolutional neural networks on lateral cephalograms from nationwide multi-centres. Orthod. Craniofac. Res..

[30] Hendrickx J., Gracea R.S., Vanheers M., Winderickx N., Preda F., Shujaat S., Jacobs R. (2024). Can artificial intelligence-driven cephalometric analysis replace manual tracing? A systematic review and meta-analysis. Eur. J. Orthod..

[31] Chen Y., Chen S.K., Yao J.C., Chang H.F. (2004). The effects of differences in landmark identification on the cephalometric measurements in traditional versus digitized cephalometry. Angle Orthod..

[32] Kazimierczak W., Serafin Z., Nowicki P., Nożewski J., Janiszewska-Olszowska J. (2024). AI in Orthodontics: Revolutionizing Diagnostics and Treatment Planning-A Comprehensive Review. J. Clin. Med..

[33] Bor S., Ciğerim S.Ç., Kotan S. (2024). Comparison of AI-assisted cephalometric analysis and orthodontist-performed digital tracing analysis. Prog. Orthod..

